# Priority use of medium-chain fatty acids during high-intensity exercise in cross-country skiers

**DOI:** 10.1186/s12970-018-0265-4

**Published:** 2018-12-10

**Authors:** A. Yu. Lyudinina, G. E. Ivankova, E. R. Bojko

**Affiliations:** 1 Department of Ecological and Medical Physiology, Ural Branch, Institute of Physiology, Russian Academy of Sciences, Pervomaiskaya av.50, Syktyvkar, 167982 Russia; 2 Medical Institute, Syktyvkar State University named Pitirim Sorokin, Starovskogo str 55, Syktyvkar, Russia; 3 Department of Ecological and Medical Physiology, Ural Branch, Head of Department, Institute of Physiology, Russian Academy of Sciences, Pervomaiskaya av.50, Syktyvkar, 167982 Russia

**Keywords:** Medium-chain fatty acids, High intensity and submaximal exercise, Competition, Cycle exercise until exhaustion

## Abstract

**Background:**

One of the topics discussed in sports science is the use of medium-chain saturated fat as an energy-saving nutrient additive when approaching high-intensity exercise. The purpose of this study was to compare the blood concentrations of medium-chain and long-chain fatty acids (FAs) across different intensity loads*.*

**Methods:**

Fifteen male highly trained athletes from the Russian cross-country skiing team participated in the study. Blood samples were drawn at rest, at the peak of veloergometric test with a growing exercise load till exhaustion (97–100% VO2max), and after competitions. The plasma FA profile was determined using gas-liquid chromatography.

**Results:**

We observed a substantial increase in the concentrations of capric acid (С10:0) (+ 164.1%), lauric acid (С12:0) (+ 223.9%), and myristic acid (С14:0) (+ 130.2%) in skiers after a sprint distance (1.3 km). A less intense increase in the concentrations of these acids (*p* < 0.05) was observed after a middle length distance or cycle exercise «until exhaustion». No significant differences in long-chain saturated FA content relative to baselines during exercise tests or competitions were revealed.

**Conclusions:**

In conclusion, the obtained results demonstrate activation of the lipolysis and the oxidation of medium-chain FA involved in the energy supply for highly trained athletes at maximum exercise loads.

## Background

Cross-country skiing is a cyclic type of sport exercising the aerobic-anaerobic capabilities of an organism. Endurance training of athletes leads to lipid mobilization and oxidation, which is evidenced by lipolysis of triglycerides (TG) to produce an increase in free fatty acids (FAs) and glycerin levels [1–3]. Although an increase in the total concentration of plasma nonesterified FAs (as a result of augmented lipolysis in adipose tissue) during such efforts is well documented, little is known about the effect of exercise on their percent distribution. Some studies [4, 5] have reported that medium intensity exercise and exercise till exhaustion changes the percentage of individual plasma FAs, notably reduced the content of the major saturated fatty acids - palmitic acid С16:0 and stearic acid С18:0, although there is no consensus on this issue.

The fatty acid (FA) oxidation increased 5- to 10-fold above resting levels during low exercise intensities (< 40% VO2max) to moderate exercise (< 70% VO2max 2) and peaked at exercise intensities around 65–70% of maximal oxygen uptake (VO2 max) [6–8]. With increasing physical load intensity, oxidation substrates switch towards carbohydrates utilization [2, 9, 10] and the oxidation of FAs gradually decreases [7, 11, 12]. A higher availability of endogenous and exogenous carbohydrates can intensify carbohydrate oxidation and reduce fat oxidation [9, 10]. However, a greater lipid supplementation at submaximal and maximal exercise is accompanied by reduced muscle glycogen breakdown. Thus, well-trained sportsmen exhibit higher fat utilization potential [13]. However, the precise mechanisms regulating the shifts in the use of oxidation substrates and the utilization of individual FAs, as well as their role in adaptation to the effects of high-intensity loads, have yet to be clarified [11, 14].

Sidossis, Coyle and their colleagues utilized stable isotope methodology to directly quantify both long- and medium-chain fatty acid oxidation during various manipulations of glycolytic flux (by infusing insulin and glucose so that glycogenolysis and glycolysis are greatly stimulated) at rest and during exercise [10, 15]. Sidossis et al. (1997) compared whole-body [13C]-labelled oleate (LC-FA) and [13C]-labelled octanoate (MC-FA) utilization during low- and high-intensity exercise. These investigators reported that oleate oxidation decreased by ~ 40%, whereas octanoate oxidation increased by ~ 30% during exercise from 40 to 80% VO_2peak_ and concluded that fatty acid oxidation is likely limited during high-intensity exercise because of direct inhibition of LC-FA (C16-C22) entry into mitochondria. In this respect, medium-chain fats (C8-C14) have been proposed as energy-saving food additives on High-Intensity Exercise [13, 16, 17], and parenteral medium-chain TG (MCT) additives during high intensity loads have been suggested to increase fat oxidation [18].

The present study primarily addresses one of the most prominent theories to explain the phenomenon of diminished fat oxidation during high intensity and submaximal exercise. A greater understanding would undoubtedly lead to novel strategies to increase fat utilization and, as such, improve exercise capacity. Moreover, no studies have investigated the blood level of medium-chain FAs (MC-FAs) in elite athletes during high intensity loads (by 90–100% VO2max) and competition.

The purpose of this study was to compare the concentrations of medium-chain and long-chain saturated fatty acids by exercise loads of varying intensity*.* We hypothesized that trained endurance athletes use MC-FAs more than LC-FAs through high-intensity exercise and that MC-FA may therefore represent an excellent energy source because they oxidize more rapidly.

## Materials and methods

### Study participants

We studied real members of national skiing team during the general training season as well as during competitions. Fifteen healthy male high-trained athletes (age: 20.7 ± 5.4 years; body mass: 68.9 ± 5.2 kg; body mass index: 22.2 ± 1.3 kg/cm2, body fat: 9.4 ± 3.1%) participated in the study. All athletes had extensive experience in endurance events and had a minimum of 5 years cross-country skiing practice as part of their main training schedule. The subjects provided written informed consent to participate in the present study. The experimental protocol was in accordance with the Declaration of Helsinki. The study was designed and performed according to the guidelines of the Local Research Bioethics Committee of the Institute of Physiology of the Komi Scientific Centre of the Ural Branch of the Russian Academy of Sciences.

### Experimental design

The study was performed during the morning; therefore, all of the volunteers consumed a standardised meal (400–420 kkal) consisting of (in units of the percentage of the total energy supplied by the entire meal, En%) 78 En% carbohydrate, 14 En% fat and 8 En% protein. Dietary intake in our participants was assessed using food frequency questionnaire. Calories of breakfast were not normalized by body weight.

The athletes performed veloergometer test until exhaustion in June 2014 during training period. Later, in November 2014 during they participated in 1.3-km ski race (sprint, high intensity exercise), and on the next day they participated in a 15-km (medium distance, medium intensity exercise) race (both in classical cross-country style) by Russian Cross-Country Skiing Cup.

We chose two distances in the same style (classic style), which differ in the degree of energy supply of muscle activity: a sprint (1.3 km) and a distance race of 15 km. As is known the remote racing is associated with work in aerobic-anaerobic, that is mixed, energy supply zone, that power is usually adequate to 8–10 mmol/L lactate, and in our case corresponded to lactate at the finish of the 15-km race 7.9 mmol/L. Energy supply occurs mainly due to aerobic oxidation of carbohydrates and anaerobic glycolysis.

**Fig. 1:**
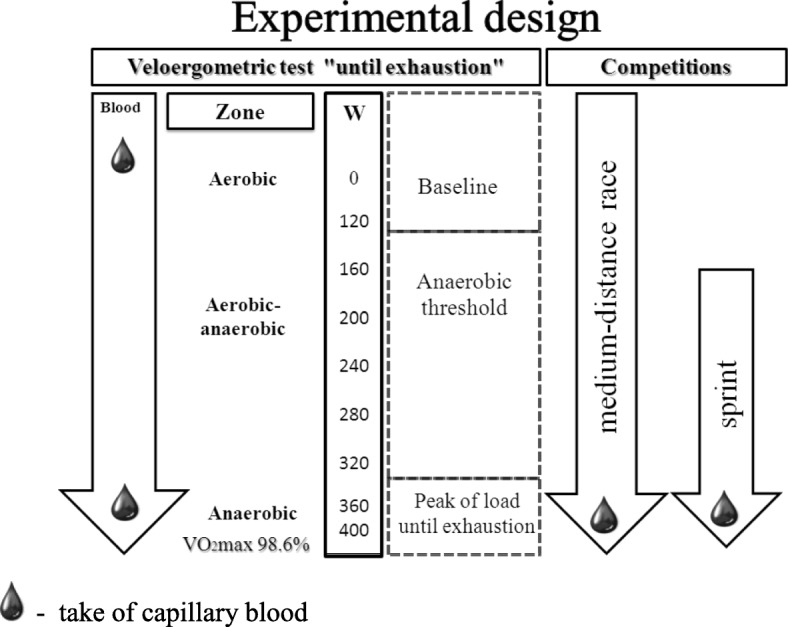
Overview of the study design of Veloergometric test «until exhaustion» and competitions

The protocol of blood sampling in the experiment is depicted in Fig. 1. Capillary blood samples were drawn at rest before exercise (baseline) and under three different conditions: immediately after exercise “until exhaustion”, 5 min after a short-distance race (1.3 km), and 5 min after a medium-distance race (15 km).

### Veloergometer test “until exhaustion”

Aerobic capacity (VO_2max_) testing was performed on an ergometer bike («Ergoselect-100», Ergoline GmbH, Germany). The protocol included one minute of cycling without load (for adaptation), then stepwise load increase by 40 W in 2 min time increments starting with initial load of 120 W. Pedalling speed was 60 rpm,. Heart rate and work load were continuously recorded, Breath-by-breath measurements (VO2 and VCO2) were taken throughout exercise by using an automated gas analysis system (Jaeger Oxycon Pro, Wuerzberg, Germany). Maximal oxygen uptake (VO_2max_) was 4.3 ± 0.4 l min_- 1_, and maximal oxygen uptake by RER (VO_2max_ RER) was 3.5 ± 0.6 l min_-1_.

Fat oxidation were determined by indirect calorimetry and plotted as a function of exercise intensity.

### Measurement of fatty acids

The plasma profile of total fatty acids was determined using gas-liquid chromatography. Sample preparation included lipid extraction from plasma [19] and obtaining FA methyl ethers using methanol and acetyl chloride, as described by Lillington et al. (1981) [20]. Gas-liquid chromatography analysis of the FA methyl ethers was performed on a gas chromatograph («Crystal 2000М» Chromatek, Russia) with a flame ionization detector attached to a SupelcoWAX (25 m × 0.23 mm) capillary column (Supelco, USA) at a temperature range from 170 °C to 250 °C (retention time, 2 min). The temperature increase rate was 4 °C/min (overall time, 25 min). Helium was used as the carrier-gas, the volume rate was 0.6 ml/min, and the flow separation rate was 1/65. The evaporator temperature was 260 °C, and the detector temperature was 200 °C. FA identification was performed using Sigma standards. The quantitative analysis of the FА concentrations was performed using the internal standard of margarine acid solution (C17:0). The FA concentrations were expressed as absolute data (mg/ml) and as a weight percentage of the total level of FAs. The reference values 1.9–4.2 mg/ml were taken as normal concentrations [21]. The reference means of plasma total FA (mol%) were taken from a literature source [22].

### Statistical analysis

Statistical analyses were performed using Statistica software (version 6.0, StatSoft Inc., 2001, USA). All results were presented as arithmetic mean values with standard deviations (M ± SD) Average ± Standard Error.The significance of variations between groups was estimated by the Wilcoxon test. The correlation coefficients between two variables were determined by Spearman rank analysis. The value *p* < 0.05 was accepted as statistically significant.

## Results

### Plasma FA at rest

According to our data, baseline values of the total pool of FA in the blood plasma of skiers during the pre-competition period were lower than the recommended values [21], with an average of 1.44 ± 0.16 mg/ml. The total FA level of capillary blood ranged from 1.1 to 2.6 mg/ml. The MC-FAs consisted of 0.17 ± 0.05% capric acid (C10:0), 0.20 ± 0.13% lauric acid (C12:0), and 1.04 ± 0.52% myristic acid (C14:0). The average value of the LC-FA palmitic acid (C16:0) was 22.7 ± 5.3%, and level of stearic acid (C18:0) was 10.0 ± 2.3%. Thus, analysis of the FA profile of the skiers in the pre-competition period revealed higher concentrations of saturated FA compared with the reference means.

A negative correlation was observed between saturated FA concentrations in total blood lipids and heart rate at rest (C 14:0 – r_s_ = − 0.619; *p* = 0.024 and C18:0 – r_s_ = − 0.587; *p* = 0.035) (Table 1).

**Table 1:** Correlation analysis between levels of saturated fatty acids in total lipids and heart rate from high-trained skiers at rest and after exercise «until exhaustion»

	at rest	after exercise «until exhaustion»
HR & C10:0	Rs = −0.156 *p* = 0.602	**Rs = − 0.564** *p* = 0.028
HR & С12:0	Rs = − 0.275 *p* = 0.362	Rs = − 0.168 *p* = 0.549
HR & C14:0	**Rs = −0.619** *p* = 0.024	**Rs = − 0.528** *p* = 0.043
HR & C16:0	Rs = −0.545 *p* = 0.054	Rs = − 0.175 *p* = 0.532
HR & C18:0	**Rs = − 0.587** *p* = 0.035	Rs = 0.005 *p* = 0.985

Note: *HR* heart rate, *Rs* correlation coefficient, *C10:0* Capric acid, *C12:0* Lauric acid, *C14:0* Myristic acid, *C16:0* Palmitic acid, *C18:0* Stearic acid

### Plasma FA after exercise until exhaustion

Table 2 presents data on oxidation of fats at rest before exercise (baseline), at anaerobic threshold and peak of load.

**Table 2:** Fat oxidation and data of RER from high-trained skiers at rest and during exercise «until exhaustion» / (Average ± Standard Error)

Test stage	RER	Fat Oxidation, g/min	VO_2_
Baseline	0.79 ± 0.02	0.20 ± 0.02	0.52 ± 0.02
Anaerobic threshold	0.98 ± 0.00	0.11 ± 0.03	3.54 ± 0.81
Peak of load “until exhaustion”	1.12 ± 0.03	0.00	4.25 ± 0.97

Note: *RER* respiratory exchange ratio, *Rs* correlation coefficient;

Fat oxidation and data of RER from high-trained skiers at rest and during exercise «until exhaustion» /M (min-max).

On increasing exercise intensity further, fat oxidation rate started to fall, and RER values of 1 (RER = 1) were reached around 84%V O2 max.

Calculated fat oxidation at the peak of load on average 330 W was zero. The test was terminated at VO_2_ max - in the range from 97 to 100%, that is, the exercise in test «until exhaustion» was maximum intensity in anaerobic zone.

**Fig. 2:**
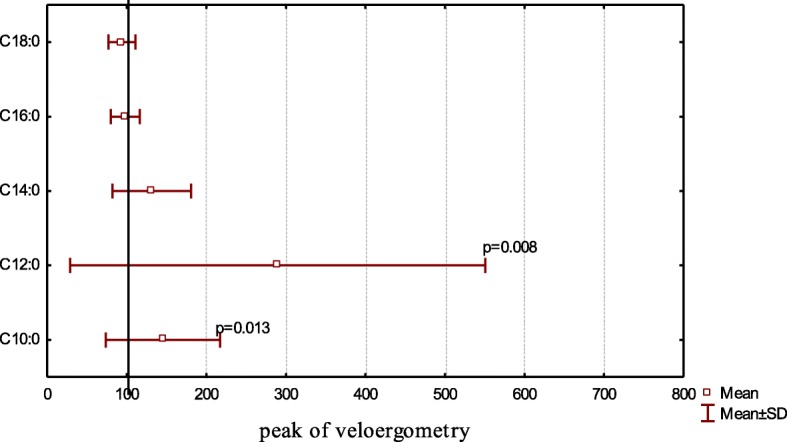
Relative saturated acid content at peak of veloergometry to compare baseline (100%)

After ergometric exercise, the average value of the total FA pool was 1.44 ± 0.25 mg/ml. No significant differences in total FAs were found between rest and peak load (*p* = 0.808). Analysis of the saturated FA profile of the skiers revealed a significant increase in the level of capric acid (C10:0) (*p* = 0.013), twice as much as the increase in lauric acid (C12:0) (*p* = 0.008) relative to baseline (Fig. 2).

No differences in the level of the myristic acid (C14:0) (*p* = 0.345), palmitic acid (C16:0) (*p* = 0.916), or stearic acid (C18:0) (*p* = 0.152) were observed between rest and peak load. A negative correlation was observed between saturated FA concentrations and heart rate after exercise until exhaustion (C10:0 – r_s_ = − 0.564; *p* = 0.028; C 14:0 – r_s_ = − 0.528; *p* = 0.043) (Table 1).

### Plasma FAs after competitions

**Fig. 3:**
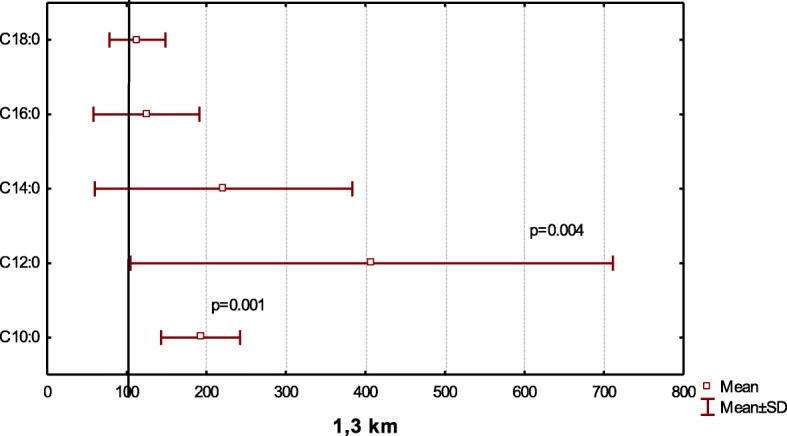
Relative saturated acid content at after competition distances 1.3 km to compare baseline (100%)

**Fig. 4:**
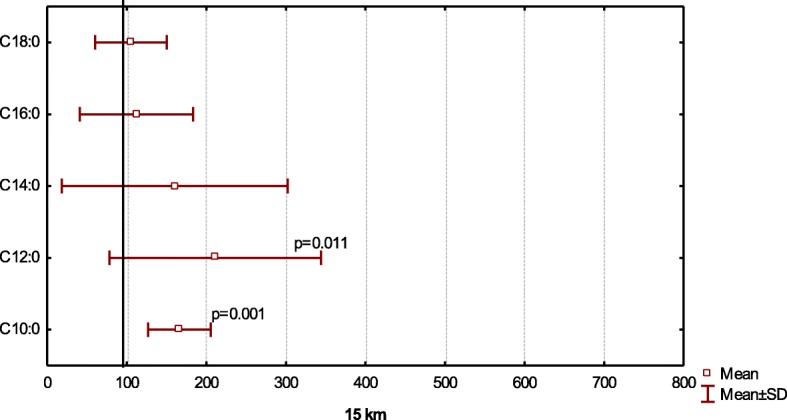
Relative saturated acid content at after competition distances 15 km to compare baseline (100%)

After a short-distance race (1.3 km), the percentage of capric acid increased from 0.17 to 0.28% (*p* = 0.001), similar to the increase after 15 km (p = 0.001) (Figs. 3, and 4). We also observed a two-fold increase in lauric acid (С12:0) content after 1.3-km (*p* = 0.004) and 15-km (*p* = 0.011) races. Furthermore, myristic acid (C14:0) concentrations increase 30% (*p* = 0.119) and 70% (*p* = 0.054) relative to baseline values after 1.3 km and 15 km, respectively. No significant differences in the concentrations of palmitic acid (С16:0) or stearic acid (С18:0) were after a short (*p* = 0.219; *p* = 0.136) or medium distance (*p* = 0.612; *p* = 0.772).

## Discussion

Generally, due to their specific mechanisms of metabolism and in vivo transportation, FAs are subdivided into short-chain (С4-С6), medium-chain (С8-С14), and long-chain (C16-C22) FAs [23]. According to literature [4, 5] capric acid, lauric acid, myristic acid, palmitic acid and stearic acid are the main fatty acid fuels for aerobic metabolism and key role in sports performance. Utilization of blood nonesterified fatty acids (NEFA) in working muscles is important for aerobic ATP synthesis during prolonged exercise of moderate intensity. Long-term, intense physical training significantly affects FA profile of plasma and erythrocyte phospholipids. Mougios et al. (2003) have found a significant increase in the percentage of the major unsaturated fatty acid, 18:1(n-9), and significant decreases in the percentages of the major saturated fatty acids, 16:0 and 18:0, at the end of exercise (exercise at 50–55% of maximal aerobic power for 1 h). Although the mechanism behind these alterations is not known, they may be result of changes in the relative uptake of these fatty acids by muscle [4]. Exercise «till exhaustion» reduced the content of palmitic acid in white musculus gastrocnemius, increased the content of myrystic and palmitoleic acid in soleus and reduced the content of oleic acid in the same muscle [5].

According to our data, baseline FA values in the capillary blood of skiers during the pre-competition period were lower than the recommended levels [21]. Endogenous triacylglycerols present in adipose tissue and skeletal muscle, FAs and their derivatives are important energetic substrates during endurance exercise. It is known that aerobic exercise (physical load) increases both lipolysis in adipose tissue and FA oxidation in muscle. Balance of these two processes determines TG and FA levels in serum in exercise. FA oxidation rate is determined by energy requirements of the working muscles, FA transport to mitochondria, and oxidation of other energy substrates. [1, 2, 12].

Adipose triglyceride lipase and hormone-sensitive lipase, which regulate lipolysis in skeletal muscles, are activated by physical loads [24].The increased use of triacylglycerol during exercise represents a careful integration of neural, hormonal, circulatory, and muscular events that increase energy requirements and facilitate delivery of fatty acids from adipose tissue and intramuscular triacylglycerols stores to skeletal muscle mitochondria for oxidation [25].

Mild- or moderate-intensity exercise [25–65% of maximal oxygen consumption (VO2max)] is associated with a 5–10-fold increase in fat oxidation above resting amounts because of increased energy requirements of muscle and enhanced fatty acid availability. A large portion of the increased supply of fatty acids is provided by lipolysis of adipose tissue triacylglycerols, which increases 2–3-fold and is mediated by increased β-adrenergic stimulation [25]. As a result, the FA pool is refilled from various sources, including FAs transported freely or combined with albumin, FA from TG components of low-density lipoproteins, and FAs formed as a result of TG lipolysis of muscle cells [11, 25]. The exercise-induced changes in the fatty acid profile of serum reflect the fatty acid profile of adipose tissue. These findings may prove useful in discovering mechanisms underlying the effects of exercise training on the fatty acid composition of human tissues [4]. During the first 120 min of exercise, the lipolytic rate is approximately twice the rate of fatty acid oxidation. Another fat source, presumably plasma or intramuscular TG, is being oxidized in addition to plasma fatty acid derived from adipose tissue [25].

Our study presents data on changes in the level of saturated blood acids in standardized conditions (bicycle ergometric test is a convenient model for studying the mechanisms of exercise load effects. In real competitions there are physiological and psycho-emotional stress factors, as well as external factors (cold, wind, competition) influence, which increases manyfold the degree of load on the body, as contrasted with the test in the laboratory.

We demonstrated that cycle ergometric test to exhaustion, and high-intensity (sprint) and medium intensity (15 km race) exercise had similar effects on serum saturated FA profile in cross-country skiers, especially with respect to MC-FA.

We observed that the level of capric acid (С10:0) (*p* = 0.013) rose considerably (60%) at cycle load (Fig. 2) and after various skiing competition distances (*p* = 0.001) (Figs. 3, and 4). We also noted a significant, twofold increase in lauric acid (С12:0) concentrations at the peak of cycle load (*p* = 0.008) and upon completion of 1.3-km (*p* = 0.004) and 15-km (*p* = 0.011) races. Furthermore, we observed a trend for increasing myristic (C14:0) acid concentrations from 30 to 70% relative to baseline values after competition (*p* > 0.05).

These data are consistent with results demonstrating that high intensity loads corresponding to 80% VO2 are accompanied by suppressed oxidation of LC-FAs but not MC-FAs [15] as well as a significant serum increase in medium-chain but not long-chain TG [13]. MC-FAs such as decanoate (capric acid, C10:0) are assumed to traverse cell and organelle membranes through non–carrier-mediated means, whereas LC-FAs (e.g., oleate (C18:1), palmitate (C16:0)) must utilize a carnitine-acyl carnitine exchange system for mitochondrial entry [26].

Previous studies investigating FA oxidation reported that MC-FAs (e.g., С12:0) oxidize faster, whereas long-chain saturated FAs (С16:0 and С18:0) oxidize more slowly [27]. MC-FAs can be transported independent of this carnitine-dependent transport system, and carnitine-mediated transport does not appear to contribute to an enhancement in MC-FA oxidation with exercise training. Although LC-FAs are transported by carnitine palmitoyl transferases, MC-FAs are transported by carnitine octanoyl transferases across the inner mitochondrial membrane [26].

According to studies by Sidossis et al. (1997) the level of fatty acids in the blood was studied under exercise loads of different intensity: the volunteers performed exercise either at 40% VO2peak for 60 min or at 80% VO2peak for 30 min. In our study, exercise load till exhaustion was adopted, blood samples was drawn at rest and at the peak at the peak of test till exhaustion, corresponding to an average of 98.6% of VO2max (in the anaerobic zone, zone 5), and not 80% VO2peak as in the article Sidossis. The test was terminated at VO2 max - in the range from 97 to 100%, that is, the exercise in test «until exhaustion» was maximum intensity in anaerobic zone.

We detected a negative correlation between serum saturated FA concentrations and heart rate at rest and after exercise until exhaustion (Table 1). We found it interesting that these relationships promote the oxidation of MC-FAs. Octanoate (C10:0) contributes 50% of the acetyl-CoA pool in the heart and soleus muscle under normal physiological conditions. Octanoate metabolism increases in contracted muscle proportional to the overall increase in the oxidative rate [28]. However, further discussions would be speculative.

The observed level of long-chain saturated FAs (palmitic C16:0 and stearic C18:0) in the blood plasma at maximum loads did not exhibit any significant changes. Previous studies reported that the profile of FAs at the end of prolonged moderate exercise is characterized by an increase in the oleic acid proportion and a decrease in serum palmitic and stearic acids concentrations [4] because saturated and monoenic FAs act as substrates for oxidation and cellular energy. However, a decrease in 16:0 FAs is in disagreement with other studies that reported no change [29].

The LC-FAs are unable to cross the mitochondrial membrane in the absence of carnitine. The LC-FAs require enzymatic transport into the mitochondrial matrix for *β*-oxidation (for fuel usage). Mitochondrial acyl co-enzyme A is subject to oxidation and yields excess acetyl co-enzyme A due to the availability and rapid oxidation of medium-chain TGs, resulting primarily in increased ketone production. Ketone bodies may act as another energy source for muscle cells and extrahepatic tissues [30]. During exercise at 85% of VO_2max_, plasma FA levels were reduced to very low levels, and this reduced fatty acid availability has been shown to reduce LC-FA oxidation [15]. Furthermore, FA utilization is inhibited [11]. The most likely mechanism underlying oxidation inhibition of common FAs at medium and high intensity loads is the low supply of free carnitine, lowering the internal cellular pH [5] and decreasing carnitine-palmitoyl-transferase I activity to inhibit transportation of long-chain FAs in mitochondria [15]. Strong correlational evidence exists that muscle free carnitine availability is likely to be a key limiting factor to fat oxidation during high intensity, submaximal exercise [8]. Thus, carnitine is considered the primary direct regulator of FA oxidation [11].

It is generally accepted that in high-intensity exercise (e.g. sprints) energy is provided mostly through anaerobic metabolism, primarily by ATP synthesis in glycolysis [3, 8]. The sprint that is 4 runs of 4 min, for the last runs the role of aerobic contribution increases due to depletion of anaerobic mechanisms, therefore a highly qualified sprinter have to have good aerobic capabilities, which, under certain conditions, makes it competitive and distance racing. In the sprint at distances with hill the anaerobic mechanism with an increase in lactate from 10 to 20 mmol/L gives 40% of energy. At the finish the level of lactate of our sprinters was averaged 12.4 mmol/L.

One of the topics discussed in sports science is the use of medium-chain saturated fat as an energy-saving nutrient additive by high-intensity exercise. It has been suggested that medium-chain triacylglycerol (MCT) ingestion could elevate the concentration of free fatty acid in plasma, reduce muscle glycogen use, and, hence, improve endurance capacity [30]. MCT ingestion in endurance-trained cyclists by exercise for 2 h at 63% of peak oxygen consumption only raised serum free fatty acid (FFA) and higher ketone body concentrations [31]. However, some authors believe that taking (long-term or one-time) MCT before training fails to decrease glycogen oxidation in muscles during high intensity exercise [25], and fails to increase stamina or alter the metabolism of runners during training [16].

The studies described above were conducted at low- to moderate-intensity exercise (60 to 70% of VO2max) [32]. Horowitz et al. (2000) [25] argued that medium-chain TG ingestion may be more effective in reducing muscle glycogen breakdown under conditions in which fatty acid availability is limiting, such as during high-intensity exercise. An increase in the *β*-oxidation of MC-FAs and lower carbohydrate utilization is also known to considerably reduce heart load during prolonged training sessions of low and medium intensity [13, 17, 33].

There are contradictory data on the effect of food MC-FA on their concentration in the blood. According to some authors, using of MC-FA during physical exercises promotes increasing MCFA in the blood [25, 31]. But only physical load leads to the same result. A number of authors consider that taking 25–30 g of MST does not affect the lipid profile of the blood [32]. In our study, breakfast did not include fat-containing products with MC-FA, because of we suppose that changes caused by exercise are due to endogenous effects on fat metabolism. In general, parenteral lipid additives during high intensity loads increase fat oxidation, but the effect of long-chain or medium-chain TGs as metabolism substrates remains to be clarified [25].

## Conclusions

The study of professional cross-country skiers at rest during the general training period revealed a decrease in the total FA pool in their blood plasma. Cycle ergometric loads «until exhaustion» and competition (1.3 and 15 km races) identically modified the serum saturated FA profile, which was manifest as a considerable increase in the concentrations of MC-FAs (capric, lauric and myristic), with no change in long-chain fatty acid concentrations relative to baseline values.

The practical aspect of this study indicates possible using of supplements containing medium chain fats in form of TG under high intensity loads (at maximum exercise loads), for example, in sprint.

## Abbreviations

BMI: body mass index
FAs: fatty acids
LC-FA: long-chain fatty acids
MC-FA: medium-chain fatty acids
MCT: medium-chain triacylglycerol
TG: triglycerides
